# The conscious crow

**DOI:** 10.3758/s13420-021-00466-5

**Published:** 2021-02-17

**Authors:** Onur Güntürkün

**Affiliations:** Department of Biopsychology, Institute of Cognitive Neuroscience, Faculty of Psychology, Ruhr University Bochum, 44801 Bochum, Germany

## Abstract

Nieder, Wagener, & Rinnert (*Science, 369*(6511), 1626–1629, 2020) demonstrated that some neurons in a prefrontal-like brain area of carrion crows signal neither the physical stimulus nor the intended action but the upcoming choice. This pattern of results implies that neural computations for consciousness can be generated by nonmammalian brains in similar ways as in primates.

Experimental scientists have long shied away from studying consciousness. Since only introspective reports could be used to determine a conscious experience, solid scientific inquiry seemed to be impossible. This has changed in the past 2 decades, and the shift was sparked by both conceptual advances and novel experimental approaches in neuropsychology and brain imaging. These studies posit that most cognitive processes can be realized at a subconscious level, while two types of cognitions require consciousness (Dehaene, Lau, & Kouider, 2017). The first is the ability to attend to a specific stimulus such that we can subsequently report its presence. The second class of cognition that is in need of consciousness is metacognition, with which we monitor our own mental processes and can reflect about them. What are the most likely neural fundaments of these abilities?

To understand the rationale of this study, we first have to introduce the global neural workspace theory. This theory assumes that the core of a cortical connectome (the “global workspace”) can, when activated, broadcast information to most nodes via long-distance connectivities (Baars, 1988). Thus, the activation of the connectomes’ core, intertwines all modules of the connectome such that they jointly participate in maintaining and processing a certain function. The global neural workspace hypothesis predicts that a stimulus first evokes an activation within a specialized perceptual module. If this local activation is able to spark activity in the core of the connectome, it will ignite a global network activity pattern that also involves higher cortical areas. As a result, this stimulus is recurrently broadcasted and becomes conscious. Thus, we should expect a two-stage process that starts with a subconscious perception and then proceeds with a broad neural activation of higher cortical areas. Only the second event should correlate with the conscious self-report of the subject.

When using these criteria, consciousness has also been shown in nonhuman primates (Dehaene et al., 2017). But does this scenario require a cortical architecture, or could it also be present in animals that have no cortex? Depending on the answer, consciousness would be confined to mammals or could constitute a broader phenomenon. A perfect group of animals to seek an answer are birds that were assumed to have no cortex, but enjoy excellent cognitive abilities that can be exploited for such a study. Therefore, Nieder, Wagener, and Rinnert (2020) tested two carrion crows (*Corvus corrone*) to study consciousness. Corvids are sometimes called “feathered apes” because their cognitive abilities are on par with those of our closest primate cousins. Indeed, Nieder et al. (2020) used a procedure that had been used in monkeys to probe for signatures of consciousness. In short, their crows played a delayed stimulus detection task. First, the birds had to initiate a trial after which a gray square appeared in 50% of the cases. A key design feature was that this visual stimulus was presented with variable intensity. Sometimes it was so bright that the crows easily detected it. In other trials, the stimulus was presented at threshold such that the animals could only detect the dim square in about half of the cases. For the birds, it was important to know whether a stimulus had been shown or not because after a delay of 2.5 s, a rule cue (blue or red) appeared. This rule cue had to be treated differently, depending on the previous presence or absence of the square stimulus. In trials in which the square stimulus had been presented, the crows had to respond to the red cue, but not to the blue one. After the absence of the square stimulus, blue and red cues had to be treated invertedly. While the crows were working on this task, single unit recordings took place from the nidopallium caudolaterale (NCL), a pallial area that constitutes the functional analogue to the mammalian prefrontal cortex (PFC).

The results showed that supra-threshold square stimuli resulted in proper responses to red and blue rule stimuli, while performance was much lower for near-threshold gray square stimuli. During task execution, 488 NCL-neurons were recorded, of which 262 were task-selective. These cells could be classified into two groups. One group of cells fired as soon as the square stimulus appeared and their activity correlated with stimulus intensity. Their correlation with the subsequent response of the birds was, however, rather low. This was different for the second group of neurons, which mainly fired during the delay period subsequent to square stimulus presentation. These cells predicted the birds’ perceptual report and not the physical stimulus. Interestingly, the activity of these delay neurons also correlated with “false alarm” choices when crows made mistakes. Thus, Nieder et al. (2020) report neural responses from the avian “prefrontal” area that do not correlate with the physical stimulus but with the animals’ report. At this point, it is important to note that this neural signal cannot represent motor preparation since the crows could not know if they will face a red or blue rule stimulus and accordingly could not prepare themselves for an upcoming response. Similar patterns of results in humans and nonprimates are taken as neural correlates of visual consciousness. So, by the same token, these findings in crows constitute empirical markers for avian consciousness.

These findings of Nieder et al. (2020) seem to refute the proposition that the cortex is a prerequisite for consciousness. However, in the same issue of *Science,* Stacho et al. (2020) published a study in which they demonstrated a cortex-like neuroarchitecture of the avian sensory pallium that is constituted by complex and iteratively repeated canonical circuits. These are embedded into stacked, horizontal neural lamina and orthogonally located vertical columns. This *avian cortex* could very likely generate computations akin to its mammalian counterpart, but is confined to sensory areas and does extend to the NCL. Although the avian cortex thus constitutes only parts of the pallium, the discovery of Stacho et al. (2020) makes it unlikely that the cortex as the computational origin of consciousness can be completely rejected. However, the global neural workspace theory, although initially formulated with a cortical architecture in mind, never explicitly stressed that a cortical architecture is required (Baars, 1988). What is required, though, is a pallial small-world connectome with a core of major hubs that are surrounded by multiple specialized modules. Indeed, birds have a pallial small-world connectome with a core that is constituted by several hubs of which the NCL is the largest (Shanahan, Bingman, Shimizu, Wild, & Güntürkün, 2013). With its core and its many surrounding modules, the avian pallial connectome is highly similar to that of mammals and possibly enables similar processing dynamics as required by the global neural workspace theory.

What are the evolutionary implications of these findings? Nieder et al. (2020) discuss two evolutionary scenarios. The first is that birds and mammals inherited the neural fundaments of consciousness from their last common ancestor that lived about 320 million years ago. The second possibility is that birds, mammals, and also further vertebrate classes independently developed the trait of consciousness based on a parallel sophistication of common vertebrate pallial connectivity patterns. Yet there is a third, even more tantalizing option. Connectomes that could produce activity patterns as postulated by the global neural workspace theory of consciousness are not confined to vertebrates, but are increasingly also found in various invertebrates. This would not necessarily imply that consciousness emerging from these neural systems enables a rich mental life similar to ours. But it could provide these animals the means to simply stay focused on a certain stimulus or goal amidst the clutter of distractors. According to this third option, consciousness that was once hailed as the ultimate triumph of human evolution could constitute in its most basic form a rather mundane cognitive process that allows diverse animal species to simply keep track of what is currently important.
